# The Significant Influence of the Neuroendocrine Component on the Survival of Patients with Gastric Carcinoma Characterized by Coexisting Exocrine and Neuroendocrine Components

**DOI:** 10.1155/2019/3671268

**Published:** 2019-03-12

**Authors:** Hu Ren, Su-Sheng Shi, Nian-Chang Wang, Xing Wang, Ying-Tai Chen, Dong-Bing Zhao

**Affiliations:** ^1^Department of Pancreatic and Gastric Surgery, National Cancer Center/National Clinical Research Center for Cancer/Cancer Hospital, Chinese Academy of Medical Sciences and Peking Union Medical College, Beijing, 100021, China; ^2^Department of Pathology, National Cancer Center/National Clinical Research Center for Cancer/Cancer Hospital, Chinese Academy of Medical Sciences and Peking Union Medical College, Beijing, 100021, China; ^3^Department of Head and Neck Surgery, Zhejiang Cancer Hospital, Hangzhou, 310000, China

## Abstract

**Background:**

Gastric adenocarcinoma patients with a neuroendocrine (NE) component are frequently observed in routine practice. Several previous studies have investigated the influence of a NE component on the survival of these patients; however, the results were inconsistent.

**Methods:**

We retrospectively investigated a consecutive series of 95 gastric adenocarcinoma patients with a NE component and 190 gastric adenocarcinoma patients without a NE component. We adopted 10%, 20%, 30%, 40%, 50%, 60%, 70%, 80%, and 90% as the cut-off proportions of the NE component, respectively, and analyzed the patients' overall survival according to the proportion of the NE component.

**Results:**

The 1-, 3-, and 5-year actual survival rates of the patients with a NE component were 90.1%, 72.3%, and 67.2%, respectively, and for those without a NE component 94.2%, 79.3%, and 75.7%, respectively. The multivariate analysis showed that the patients with NE components >70% (HR: 2.156; 95% CI: 1.011, 4.597;* p=0.047*) and >90% (HR: 2.476; 95% CI: 1.088, 5.634;* p=0.031*) had significantly worse survival than those without a NE component. Only the diameter of tumors (>4.64 cm) (HR: 2.585; 95% CI: 1.112, 6.006;* p=0.027*) and pN3 (HR: 2.953; 95% CI: 1.051, 8.293;* p=0.040*) were independently associated with worse overall survival for gastric adenocarcinoma patients with a NE component (all* p<0.05*).

**Conclusion:**

Gastric adenocarcinoma patients with a NE component >70% and >90% have significantly worse survival than those without a NE component. Only the diameter of tumors and the number of metastatic lymph nodes are independent prognostic factors for gastric adenocarcinoma patients with a NE component.

## 1. Introduction

The first description of gastrointestinal tumors with exocrine and neuroendocrine (NE) components was published by Cordier in 1924 [1]. Since then, several cases have been reported with many different names including composite carcinoid, mucin-producing carcinoid, argentaffin cell adenocarcinoma, goblet cell carcinoid, adenocarcinoid, and small cell undifferentiated carcinoma. These different names led to considerable confusion among clinicians, surgeons, gastroenterologists, and pathologists [2]. The spectrum of the carcinoma shows mixed divergent differentiation along the exocrine and NE systems, and these two components express variable proportions ranging from 1% to 99% [3].

The prognostic significance of the NE component remains controversial in gastric adenocarcinoma (GAC) patients with a NE component. In 1987 [4], Lewin defined the criteria for determining the extent of the NE component in mixed adenoneuroendocrine carcinomas (MANEC) as 30%, which incidentally is the same as that currently used [3]. The cut-off proportion of 30%, however, is somewhat arbitrary, because not enough data are available for demonstrating the prognostic significance of the NE component [5]. Chen et al. [3] found that a high NE component (>50%) in primary tumors was associated with poor prognosis. Park et al. [5] suggested that a NE component (≥10%) is an independent factor for poor prognosis. But it remains unclear how the NE phenotype may confer adverse prognosis; even the NE expression might promote tumor cells' growth via an autocrine or paracrine loop [6, 7].

Our study aimed to provide a general understanding of the prognostic influence of the NE components on GAC with a NE component. To the best of our knowledge, this cohort is one of the largest to date in the literature for the patients with gastric carcinoma (GC) with exocrine and NE components.

## 2. Materials and Methods

### 2.1. Patients

A total of 95 patients with GAC with a NE component (GAC with neuroendocrine differentiation (NED), MANEC, and neuroendocrine carcinoma (NEC)) who underwent radical gastrectomy with D2 lymphadenectomy at the China National Cancer Center between February 2011 and January 2016 were identified and included in the study. To evaluate the prognostic significance of the NE component, we selected double GAC patients without a NE component during the same period according to the baseline clinicopathological factors in the group of GAC with a NE component.

The study inclusion criteria were as follows: (1) the diagnosis of GAC patients with a NE component was confirmed by two pathologists, using the 2010 World Health Organization (WHO) classification of gastric neuroendocrine neoplasms (NENs) for histopathologic evaluation [9]; (2) the patients underwent gastrectomy with D2 lymphadenectomy and R0 resection, which was determined by no macroscopic or microscopic residual carcinoma; and (3) all the data of the patients were available in terms of medical history, record of surgery, pathological report, and follow-up. The exclusion criteria were as follows: (1) patients had distant metastasis; (2) patients underwent palliative surgery; (3) patients had a total well differentiated neuroendocrine tumor (NET) (G1, G2, and G3); (4) patients died from other reasons of unexpected outcomes; (5) patients had suffered from other malignancies before GC; or (6) patients were lost to follow-up.

All the patients included in this study were examined with upper gastrointestinal tract endoscopy and enhanced CT/MRI scanning to avoid possible missing lesions. Their clinical information was obtained from their medical records in each case. All the study procedures were approved by the Institutional Review Board at the China National Cancer Center.

### 2.2. Definition and Evaluation of NE Component

The pathological conditions of all the patients were reviewed individually by two pathologists to redefine the proportion of the NE component with reference to the area of tumor cells. In the primary tumors, the NE component was confirmed with morphology and positive immunohistochemical (IHC) staining with one of three NE markers from Beijing Zhongshan Golden Bridge Biotechnology Co. Ltd. (ZsBio), China (synaptophysin SYN (cat. no. ZA-0506), chromogranin A CgA (cat. no. ZM-0076), and CD56 (cat. no. ZM-0057)). We used the archived specimens for IHC analysis with the enhanced labeled polymer system (ELPS) and divided those results into different grades according to the number of positive cells and the intensity of positive staining. The Ki-67 index was assessed in areas of highest nuclear labeling, the so-called hot spots, by the manual counting of 500-2000 cells [10, 11]. And the mitotic index per 10 high power fields (HPF) = 2 mm^2^ was counted in 50 high power fields including hot spots [11]. The scoring system for IHC was modified and used with permission by the Clinical and Laboratory Standards Institute (CLSI), document I/LA28-A2 [8].

The pathologic criteria for the NE component according to the 2010 WHO classification were as follows: (1) organoid architectures such as solid nests, sheets, broad trabeculae, or rosette formation; (2) nuclear features manifested by hyperchromatic nuclei with finely to coarsely granular, but evenly distributed, chromatin; and (3) cytoplasmic features with a scant to moderate amount of slightly eosinophilic, finely granular cytoplasm and indistinct cellular membranes [5, 9]. And the scattered positive NE cells identified in the GAC area qualified for this definition. Patients with total well differentiated NET were excluded in our study.

After evaluating the proportion of the NE component, we analyzed its prognostic influence on GAC with the NE component separately by setting 10%, 20%, 30%, 40%, 50%, 60%, 70%, 80%, and 90% as the thresholds, respectively.

### 2.3. Surgical Procedures and Follow-Up

All the patients systematically underwent gastrectomy with standard D2 lymphadenectomy performed by experienced surgeons following the Japanese Gastric Cancer Association (JGCA) guidelines [12]. The surgical gastrectomy procedures (subtotal or total gastrectomy or combined organ resection) were chosen after discussion by the multidisciplinary team based largely on the GC treatment guidelines of the JGCA. The patients were examined on a weekly basis during the period of treatment. After completion of the treatment, they were followed up every 3 or 6 months until death. The follow-up analysis was carried out on their postoperative treatment information, time to recurrence, and time of death. The long-term prognostic data were obtained from the patients' clinical records or contact with the patients' relatives by telephone.

### 2.4. Statistics

The following clinical and pathological data were collected: demographic information (gender and age), clinical and pathological tumor features (location, diameter, Bormann's classification, the Lauren classification, and classification of pT stages, pN stages, and pTNM stages according to the 7th edition of the American Joint Committee on Cancer/Union Internationale Contre le Cancer (AJCC/UICC) for GC [13]), proportion of the NE component, NE markers (SYN, CgA, and CD56), mitotic figures and Ki-67 which only refer to the NE component in this carcinoma, neoadjuvant therapy, and adjuvant chemotherapy.

As the primary endpoint, overall survival (OS) was defined as the time from surgery to death or of last follow-up (updated on March 1, 2018). The dates of death from any cause were obtained from the medical records and the China National Citizen Identity Information Center. The survival curves were analyzed with the Kaplan–Meier method and their differences were estimated with a log-rank test. The univariate and multivariate Cox proportional hazard analysis was performed to evaluate the prognostic significance for the OS, with the hazard ratio (HR) and 95% confidence interval (95% CI) generated at the same time. Some mixed factors, such as gender, age, and adjuvant therapy (yes or no), which may influence the survival in the univariate analysis, were also included in the Cox proportional hazard model (multivariate analysis). A* p* value<0.05 was considered to indicate statistically significant differences for all tests. All the tests were 2-sided and were performed using SPSS 25.0 (SPSS Inc., Chicago, IL, USA).

## 3. Results and Discussion

### 3.1. Clinicopathological Features

**Figure 1:**
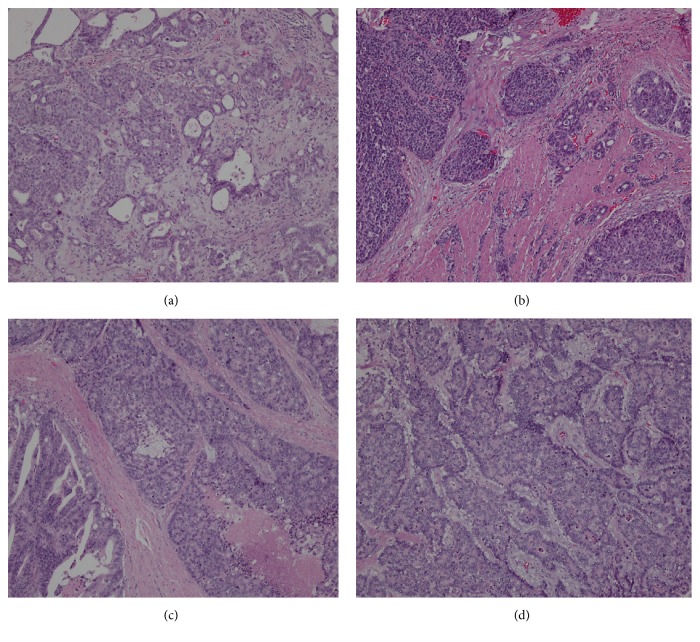
The representative pathologic images of each group (H&E X100). (a) is an emblematic image in the GAC group without a NE component; (b) is an emblematic image in the GAC group with NED; (c) is an emblematic image in the MANEC group; (d) is an emblematic image in the NEC group.

A total of 95 consecutive GAC patients with a NE component were identified, and 190 GAC ones without a NE component were included in the study. We were able to categorize the patients with GAC with a NE component into three groups according to the 2010 WHO classification of NENs of the digestive system: (1) GAC with NED (0<NE<30%) (n=32), (2) MANEC (30%≤NE≤70%) (n=29), and (3) NEC (70%<NE<100%) (n=34). There was no significant difference in gender, age, tumor diameter, tumor location, pathological status, SYN, CgA, CD56, and adjuvant therapy among these three groups, and all the clinicopathological features were not significantly different between GAC groups with and without a NE component (Table 1). The representative pathologic images of each group were shown in Figure 1.

These 95 GAC patients with a NE component, including 82 male and 13 female ones, had a median age of 61.37 (range: 39–79 years). According to the surgical records, the primary tumor preferred more to locate in the upper part of the stomach (54.74%), while 25 lesions were located in the lower part of the stomach (26.31%), and 18 lesions were in the middle of the stomach (18.95%). The median diameter of the primary tumors was 4.64 cm. According to the 7th AJCC/GC TNM staging system for GC [13], the postoperative pathological results showed that only 20 patients (21.05%) had tumors confined in the mucosa, submucosa, or muscularis propria (pT1 or pT2), while 75 patients (78.95%) had ones that invaded beyond the muscularis propria (pT3 or pT4). The status of the metastatic lymph nodes showed that only 29 patients (30.53%) were pN0 and 66 (69.47%) were pN+. Thus, most of the patients (81/95, 85.26%) had advanced diseases in the final pTNM stage. Most of the cases showed the frequent mitoses with an average of 37.71/2 mm^2^ (range: 3 to 180/2 mm^2^). And most of the cases (82/87) presented a high Ki-67 index (>20%), while only 5 presented a low one (≤20%). The mean Ki-67 index was 56.21%. Table 1 also summarized the information about the Lauren type, neoadjuvant therapy, adjuvant therapy, and relapse or metastasis.

### 3.2. IHC Staining Pattern of GAC with a NE Component

For the IHC staining pattern of these 95 cases, all the tumors were positive for at least 1 of three conventional NE markers: SYN, CgA, and CD56. The immune positivity of each NE marker was listed in Table 1. SYN was found to be the most sensitive marker which was positive in 87 cases (87/95, 91.58%), followed by CgA and CD56 which were positive in 70 cases (70/95, 73.68%) and 42 cases (42/76, 53.85%), respectively. The expression for all three markers was recognized in 22 cases (23.16%), but 2 lesions expressed SYN and/or CgA (97.89%). There were significant differences in the expressions of these IHC markers (*p<0.05*). SYN was the most sensitive marker of the NE component, while CD56 was the most insensitive marker.

### 3.3. Prognostic Significance of NE Component in GAC

**Figure 2:**
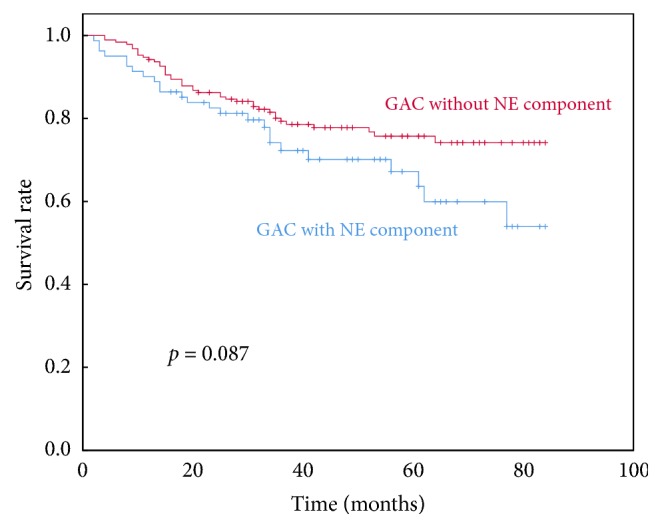
Overall survival of all the patients in the two groups.

To validate the prognostic significance of the NE component, we compared the prognoses of GAC with and without a NE component first. During the follow-up period, in the group of GAC with a NE component, 49 patients (51.58%) had recurrence or metastasis, while, in the group of GAC without a NE component, 53 (27.89%) patients did. The final follow-up results showed that the 1-, 3-, and 5-year actuarial survival rates of the patients with a NE component were 90.1%, 72.3%, and 67.2%, respectively, and for the patients without a NE component 94.2%, 79.3%, and 75.7% (Table 3). Recurrence or metastasis was more frequently identified in the patients with a NE component (*p<0.05*). However, GAC patients with a NE component (without well differentiated NET) had slightly worse survival than GAC patients without a NE component, rather than statistically significant difference (*p=0.087*) (Figure 2 and Table 2).

**Figure 3:**
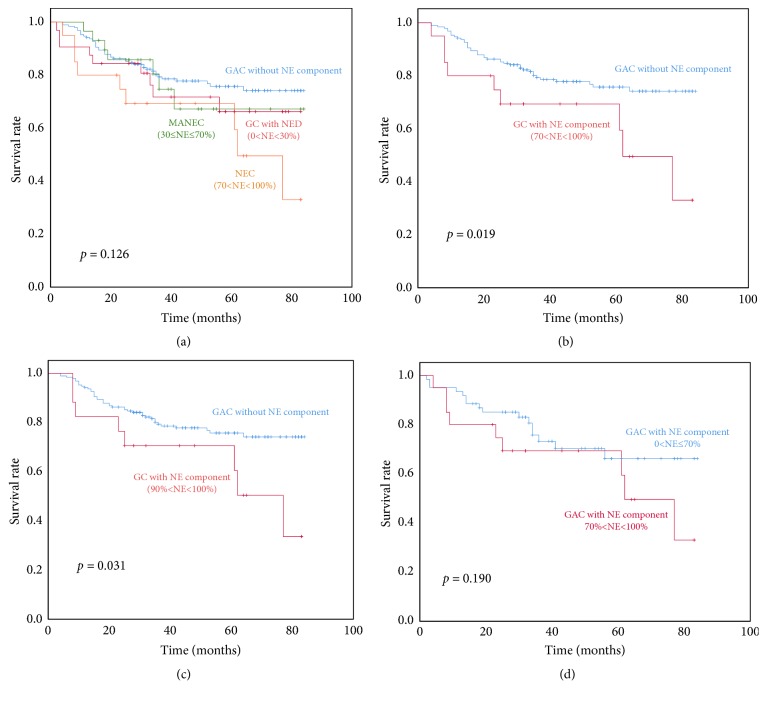
Overall survival of the patients regrouped according to the proportion of the NE component. (a) All the cases were categorized into four groups: GAC without a NE component (n=190), GAC with NED 0<NE<30% (n=32), MANEC 30%≤NE≤70% (n=29), and NEC 70%<NE<100% (n=34); there was no significant difference in OS rates among these four groups (*p=0.126*). We adopted 70% (b) and 90% (c) of the NE component as cut-off proportions, respectively; GAC patients with NE components >70% and >90% had significantly worse survival than GAC ones without a NE component (*p=0.031* and* p*=0.019, respectively). (d) We adopted 70% of the NE component as the cut-off proportions to compare the survival between two groups in GAC patients with a NE component, and there was no significant difference between the two groups (*p=0.190*).

To explore the prognostic significance of the proportion of the NE component in GAC patients with a NE component, we divided the patients with GAC with a NE component into three groups according to the 2010 WHO classification of NENs of the digestive system: (1) GAC with NED, (2) MANEC, and (3) NEC. The Kaplan–Meier survival curves of the four groups were shown in Figure 3(a) (*p=0.126*). As shown in Table 3, only NEC patients had worse outcome compared with GAC ones without a NE component in the univariate analysis (*p=0.022*). Then, we included some factors (age, tumor location, tumor diameter, and pT and pN stage) which may affect the survival of all GAC patients in the univariate and multivariate analysis (the data were not shown). The result showed that the patients with NEC still had significantly worse survival than those with GAC without a NE component (*p=0.047*).

Further, we adopted 10%, 20%, 30%, 40%, 50%, 60%, 70%, 80%, and 90% of the NE component as cut-off proportions, respectively, and analyzed the patients' OS according to the proportion of the NE component shown. As shown in Table 3 and Figure 3, the GAC patients with NE components >70% and >90% had significantly worse survival than GAC ones without a NE component in the univariate and multivariate analysis.

### 3.4. Univariate and Multivariate Analysis for GAC Patients with a NE Component

All the patients' follow-up information was available, with the median follow-up period of 44.07 months (range from 2 to 84 months) for all of them. We evaluated some possible factors associated with OS in the GAC patients with a NE component. The univariate analysis showed that the lower location in the stomach (hazard ratio (HR): 0.210; 95% confidence interval (CI): 0.049, 0.897;* p=0.035*), the diameter of the primary tumor (>4.64 cm) (HR: 3.076; 95% CI: 1.356, 6.976;* p=0.007*), and pN3 (HR: 3.359; 95% CI: 1.198, 9.417;* p=0.021*) were the significant predictors of survival. While the patients with a NE component 0<NE≤70% had longer medium survival time (64.69 months) than ones with a NE component 70%<NE<100% (56.31 months), this did not reach statistical significance (Figure 3(d)) (*p=0.190*). Other factors, such as gender, age, pT stage, pTNM stage, Ki-67, mitosis, and adjuvant therapy, were not significant predictors of survival (all* p>0.05*) (Table 4).

**Figure 4:**
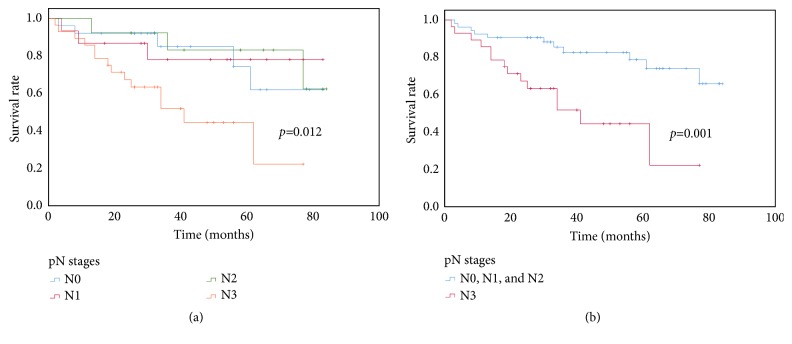
Overall survival of the patients in the GAC group with a NE component regrouped according to the pN stage. A statistically significant difference in survival was observed between patients with less metastatic lymph nodes (pN0, pN1, and pN2) and those with the number of metastatic lymph nodes exceeding 6 (pN3) (*p*<0.001). There was no significant difference in the predictors of survival among pN0, pN1, and pN2 (*p*=0.964) (the figure is not shown) (Kaplan–Meier and log-rank test).

In the multivariate analysis, only the diameter of the tumors (>4.64 cm) (HR: 2.585; 95% CI: 1.112, 6.006;* p=0.027*) and pN3 (HR: 2.953; 95% CI: 1.051, 8.293;* p=0.040*) were independently associated with worse OS (all* p<0.05*) (Table 4). Moreover, there was no significant difference in the predictors of survival among pN0, pN1, and pN2 (Figure 4).

## 4. Discussion

Only a few reports have evaluated the prognostic significance of the NE component of GC; even the coexistence of the NE component and adenocarcinoma is frequently observed [6, 14–16]. Several previous studies have proved that the well differentiated NET had better survival than the poorly differentiated NEC [17]. In this study, we included the poorly differentiated NEC to compare with other malignant tumors, such as GC, GAC with NED, and MANEC. To the best of our knowledge, this is one of the largest studies to provide a general understanding of the prognostic influence of the NE component in patients with GAC.

The most notable finding of our study was that GAC patients with a NE component >70% (NEC) and with a NE component >90% had significantly worse survival than GAC ones without a NE component. The recent 2010 WHO classification classifies NENs of the stomach into three categories according to the proportion of the NE component: MANEC (30%≤NE≤70%), a neuroendocrine tumor (NET) or NEC (NE>70%), and GC with NED (0<NE<30%) [9]. Several studies reported that the cut-off proportion of 30%, however, was somewhat arbitrary, because not enough data are available for demonstrating the prognostic significance of the NE component [5]. The previous observation reported that there was no significant difference in survival between NEC and MANEC [18, 19], although this result needs to be verified in other independent patient groups.

Firstly, we evaluated the influence of the NE component between GAC patients with and without a NE component. Several studies have reported that patients with gastric NEC had worse OS than those with GAC [6, 19]. Similarly, in our data, the OS rate of the patients with gastric NEC was poorer than that of ones with GAC without a NE component. For all GAC patients with a NE component (without well differentiated NET) in our study, the OS rates of the GAC groups with and without a NE component showed slight difference, instead of statistically significant difference (*p=0.087*), while the result in the present study showed that GAC patients with a NE component (0<NE≤70%) (GAC with NED and MANEC) had no significantly different survival compared to GAC ones without a NE component (*p=0.390*).

Then we analyzed the patients' outcomes according to the different cut-off proportions of the NE component. Several studies have reported that a higher NE component in primary tumors predicted poor prognosis in terms of GAC with a NE component [3, 5, 6]. Jiang et al. [6] suggested that when >20% SYN/CgA positivity was set, the prognosis of large cell neuroendocrine carcinomas (LCNEC) was significantly worse than that of GC with NED. But when the threshold for LCNEC was set to >50%, >40%, or >30% CgA/SYN positivity, the survival difference was not significant between the 2 groups. Park et al. [5] suggested that NEC, MANEC, and GC with NED (>10% NE) showed poorer outcomes than GC with NED (<10% NE) or without a NE component. However, it was easy to find that there was no significant difference in the survival rates among NEC, MANEC, and GC with 10–30% of NED in Park's study [20]. Furthermore, Chen et al. [3] proposed that a high NE component (>50%) in the primary tumors was associated with poor prognosis. But their study was a small sample test, because only 21 patients were included. While the mechanisms underlying this phenomenon remained unclear, a possible explanation was that the NE component might actually upregulate the expression of the vascular endothelial growth factor (VEGF) and affect the incidence of lymph node metastasis to promote neoangiogenesis [3, 21, 22]. As such, we adopted 10%, 20%, 30%, 40%, 50%, 60%, 70%, 80%, and 90% of the NE component as thresholds, respectively, and compared the patients' OS with that of the GAC patients without a NE component. When the proportion of the NE component was >70% and >90%, the patients had worse prognosis than GAC ones without a NE component. And in our research, there were only 6 GAC patients with a NE component (70%<NE≤90%), which may have influenced the result when we adopted 80% of the NE component as the threshold.

Finally, in the current research, we observed that the diameter of the tumors (≥4.64 cm) and pN3 were independently associated with worse OS for the GAC patients with a NE component, which agreed with the previous reports [19]. Park et al. [5] stated that, in the univariate analysis, larger tumor size (>4 cm) and advanced pTNM stage group were poor prognostic factors for both RFS and OS of GC. Ishida et al. found a better outcome of gastric NEC among patients with a larger tumor (>5.0 cm) (*p<0.01*) [18]. In our study, pT and pTNM stages were not significant predictors of survival, which was different from the previous reports [23]. This discrepancy might have been generated because of the fact that most of the patients were in the advanced stage of GAC in our study. According to the previous results, we set 70% of the NE component as the threshold to analyze the influence of the proportion of the NE component in GAC patients with a NE component. Possibly due to the limited sample sizes, there was no significant difference in survival between patients with a smaller NE component (0<NE≤70%) and a larger NE component (70%<NE<100%) (*p = 0.190*).

In this research, our data indicated that the prognosis of the GAC patients with a NE component and the number of metastatic lymph nodes ≤ 6 (pN1 and pN2) were similar to those of ones without metastatic lymph node involved (pN0), which was significantly better than that of the patients with the number of metastatic lymph nodes exceeding 6 (pN3). As we have known, nodal involvement is one of the most crucial indicators of prognosis in patients with resectable malignant gastric tumors following curative surgery [24]. The number of metastatic lymph nodes is a powerful prognostic factor in several malignant tumor types such as carcinoma of the digestive system and the breast [25, 26]. The classification of metastatic lymph nodes in gastric NEC is still under extensive evaluation [27]. Accordingly, we divided all patients into four groups (pN0, pN1, pN2, and pN3) according to the 7th AJCC/GC TNM staging system for GC [13].

Generally, it has been suggested that angioinvasion, clinicopathological type, mitosis, and Ki-67 index are the predictors of tumor malignancy and patients' outcome [28]. However, in our study, we found from the Cox proportional hazard model assessment that Ki-67, mitosis, and adjuvant therapy were not significant factors for prognosis of GAC with a NE component (*p>0.05*), which is inconsistent with previous studies [19, 29]. Milione et al. [30] and Boo et al. [31] reported that the Ki-67 labeling index was related to gastric NEC recurrence and prognosis. Xie et al. [19] revealed significantly reduced postrecurrence survival (PRS) for the high Ki-67 (≥57.5%) labeling index group compared with the low Ki-67 labeling index group and that the Ki-67 labeling index was an independent factor influencing the PRS of gastric NEC. Similarly, in our data, we found patients with the high Ki-67 (>56.21%) index group had slightly poorer survival than the low Ki-67 index group, rather than statistically significant difference (*p=0.097*). Moreover, according to the stratified analysis, PRS in the gastric NEC group was similar between patients who had received chemotherapy and those who had not [19]. Kubota et al. [16] found no beneficial effect of adjuvant chemotherapy for gastric NEC while Huang et al. [32] disagreed with them. One potential explanation for this conflicting result is that optimal therapy for gastric NEC remains to be established yet [33].

Few studies in the literature have reported the genetic issue of these gastric carcinomas and most have described different findings and controversial data, thus leaving various histogenetic hypotheses still unconfirmed. These gastric carcinomas may result from either the proliferation of stem cells capable of differentiating along multiple cell lineages or the simultaneous proliferation of multiple cell lineages [4, 34–38]. Scardoni et al. [35] started a mutational survey on a series of six gastroenteropancreatic MANEC to understand the molecular basis of MANEC carcinogenesis and lineage commitment. And the results showed that five of six MANEC presented similar molecular profiles in both components, which suggested an origin from a common progenitor cell of these carcinomas. Bakkelund et al. [39] found that a significant proportion of gastric cancers with signet ring cells occurring in the oxyntic mucosa were of NE origin. At least a portion of these seemed to be derived from the enterochromaffin-like (ECL) cells. Similarly, Bartley et al. [38] reported that the concordance of E-cadherin staining patterns between the signet ring and NE components may support the hypothesis that composite tumors arise from a common stem cell or precursor cell with bidirectional or multidirectional differentiation. However, it is noteworthy that Furlan et al. [36], while studying clonality of a rectal endocrine-exocrine collision tumor, found different origins of the tumor components. Although rare, the since synchronous but remote gastric tumors have been reported [40–42]. The origin of two cells in these gastric carcinomas was controversial, and we are going to start some studies in these fields in the future.

Strengths and limitations should be considered when the study results are interpreted. First, to the best of our knowledge, this is one of the largest studies to investigate the influence of the NE component on the survival of GAC patients. Second, the diagnosis of all patients was reviewed by two independent pathologists, which minimized potential disease misclassification. The major limitation of our study was the lack of the data on long-term follow-up. Moreover, the retrospective nature of this study can be associated with selection bias as well as increased risks of differential misclassification bias. In addition, all the patients analyzed were from a single institution, so the findings may not be generalizable for other settings. Also, while we had one of the largest numbers of patients to date, the sample size was modest; therefore chance cannot be ruled out for some of the significant findings.

## 5. Conclusions

In conclusion, our study found that GAC patients with a NE component >70% (NEC) and with a NE component >90% had significantly worse survival than GAC ones without a NE component. And only the diameter of tumors and the number of metastatic lymph nodes were independent prognostic factors for GAC patients with a NE component. However, the results in this study need to be further replicated in studies with larger sample sizes.

## Figures and Tables

**Table 1:** Clinicopathological features for all patients.

Variables		GAC with NE component				GAC without NE component	*p* value
		GAC with NED (0<NE<30%)	MANEC (30≤NE≤70%)	NEC (70<NE<100%)	Total		
Gender	Male	28	24	30	82	142	0.247
	Female	4	5	4	13	48	
Age (years)	≤60	15	12	16	43	100	0.241
	>60	17	17	18	52	90	
Location	Upper	19	15	18	52	86	0.127
	Middle	2	8	8	18	31	
	Lower	11	6	8	25	73	
Curvature	Lesser	16	8	14	38	91	0.194
	Greater	1	3	5	9	37	
Diameter (cm) (mean)		4.82	4.09	5.16	4.64	4.78	
Lauren type	Intestinal	15	11	4	30	63	0.234
	Diffuse	4	7	5	16	59	
	Mixed	11	8	3	22	47	
pT stage	T1	3	5	1	9	27	0.062
	T2	5	4	2	11	20	
	T3	17	14	26	57	85	
	T4	7	6	5	18	58	
pN stage	N0	11	8	10	29	59	0.462
	N1	5	7	5	17	46	
	N2	6	2	9	17	36	
	N3	10	12	10	32	49	
pTNM stage	I	5	7	2	14	35	0.717
	II	11	6	12	29	58	
	III	16	16	20	52	97	
Synaptophysin	Negative	6	2	0	8		N/A
	1+	26	15	14	55		
	2+	0	12	20	32		
Chromogranin A	Negative	6	9	10	25		N/A
	1+	26	14	14	54		
	2+	0	6	10	16		
CD56	Negative	19	11	12	42		N/A
	1+	6	4	5	15		
	2+	1	4	3	8		
	3+	0	5	6	11		
Mitotic index (2 mm^2^) (mean)		35.67	29.50	49.38	37.71		
Ki-67 (%) (mean)		56.00	55.44	57.70	56.21		
Neoadjuvant therapy	No	31	25	34	90	179	0.856
	Yes	1	1	3	5	11	
Adjuvant therapy	No	10	6	15	31	45	0.074
	Yes	18	22	18	59	141	
Follow-up (month) (mean)		43.6	38.2	40.1	40.8	45.7	

N/A: not applicable. The scoring system for IHC modified and used with permission by the Clinical and Laboratory Standards Institute (CLSI), document I/LA28-A2 [8].

**Table 2:** Overall survival rates of the two groups.

OS rate	1-year, % (95% CI)	2-year, % (95% CI)	3-year, % (95% CI)	4-year, % (95% CI)	5-year, % (95% CI)
GAC with NE component	90.1 (83.6,96.6)	82.5 (74.3,90.7)	72.3 (61.7,82.9)	70.1 (58.9,81.3)	67.2 (55.2,79.2)
GAC without NE component	94.2 (90.9,97.5)	86.3 (81.4,91.2)	79.3 (73.2,85.4)	77.8 (71.5,84.1)	75.7 (69.0,82.4)

**Table 3:** Cox regression model for the NE component.

NE component	Number	Univariate analysis		Multivariate analysis	
		HR (95% CI)	*p* value	HR (95% CI)	*p* value
No	190	1 (reference)		1 (reference)	
Yes	95	1.534 (0.935, 2.518)	0.090	1.292 (0.771, 2.164)	0.330
No	190	1 (reference)		1 (reference)	
GC with NED (0<NE<30%)	32	1.358 (0.661, 2.791)	0.404	1.224 (0.588, 2.549)	0.590
MANEC (30%≤NE≤70%)	29	1.209 (0.543, 2.695)	0.642	0.897 (0.392, 2.054)	0.797
NEC (70%<NE<100%)	34	2.318 (1.128, 4.766)	0.022	2.155 (1.011, 4.593)	0.047
No	190	1 (reference)			
GC with NE component 0<NE≤10%	30	1.429 (0.696, 2.938)	0.331		
GC with NE component 10%<NE<100%	65	1.601 (0.0899, 2.849)	0.110		
No	190	1 (reference)			
GC with NE component 0<NE≤20%	32	1.358 (0.661, 2.791)	0.405		
GC with NE component 20%<NE<100%	63	1.656 (0.930, 2.946)	0.086		
No	190	1 (reference)		1 (reference)	
GC with NE component 0<NE≤30%	42	1.131 (0.568, 2.256)	0.726	1.067 (0.530, 2.151)	0.855
GC with NE component 30%<NE<100%	53	2.013 (1.116, 3.633)	0.020	1.524 (0.816, 2.847)	0.186
No	190	1 (reference)		1 (reference)	
GC with NE component 0<NE≤40%	52	1.311 (0.716, 2.402)	0.380	1.172 (0.634, 2.167)	0.614

GC with NE component 40%<NE<100%	43	1.959 (1.008, 3.806)	0.047	1.513 (0.749, 3.059)	0.249
No	190	1 (reference)			
GC with NE component 0<NE≤50%	54	1.379 (0.764, 2.487)	0.286		
GC with NE component 50%<NE<100%	41	1.847 (0.927, 3.683)	0.081		
No	190	1 (reference)			
GC with NE component 0<NE≤60%	58	1.169 (0.769, 2.435)	0.286		
GC with NE component 60%<NE<100%	37	1.955 (0.951, 4.018)	0.068		
No	190	1 (reference)		1 (reference)	
GC with NE component 0<NE≤70%	61	1.289 (0.725, 2.294)	0.388	1.060 (0.585, 1.920)	0.848
GC with NE component 70%<NE<100%	34	2.318 (1.128, 4.765)	0.022	2.156 (1.011, 4.597)	0.047
No	190	1 (reference)			
GC with NE component 0<NE≤80%	65	1.368 (0.778, 2.404)	0.276		
GC with NE component 80%<NE<100%	30	2.070 (0.971, 4.412)	0.059		
No	190	1 (reference)		1 (reference)	
GC with NE component 0<NE≤90%	67	1.334 (0.759, 2.344)	0.317	1.061 (0.592, 1.902)	0.842
GC with NE component 90%<NE<100%	28	2.253 (1.057, 4.802)	0.035	2.476 (1.088, 5.634)	0.031

**Table 4:** Univariate and multivariate analyses for patients with a NE component.

Variables		Univariate analysis		Multivariate analysis	
		HR (95% CI)	*p* value	HR (95% CI)	*p* value
Gender	M	1 (reference)			
	F	0.828 (0.248, 2.770)	0.760		
Age	≤60	1 (reference)			
	>60	2.310 (0.964, 5.536)	0.061		
Location	Upper	1 (reference)		1 (reference)	
	Middle	0.278 (0.065, 1.190)	0.084	0.338 (0.074, 1.551)	0.163
	Lower	0.210 (0.049, 0.897)	0.035	0.391 (0.083, 1.848)	0.236
Curvature	Lesser	1 (reference)			
	Greater	1.703 (0.349, 8.321)	0.511		
Diameter	≤4.64	1 (reference)		1 (reference)	
	>4.64	3.076 (1.356, 6.976)	0.007	2.585 (1.112, 6.006)	0.027
Lauren type	Intestinal	1 (reference)			
	Diffuse	0.941 (0.289, 3.068)	0.920		
	Mixed	1.092 (0.406, 2.938)	0.861		
pT stage	1	1 (reference)			
	2	0.619 (0.039, 9.922)	0.734		
	3	1.775 (0.233, 13.527)	0.580		
	4	3.297 (0.405, 26.867)	0.265		
pN stage	0	1 (reference)		1 (reference)	
	1	0.934 (0.223, 3.915)	0.926	0.952 (0.226, 4.021)	0.947
	2	0.827 (0.196, 3.486)	0.796	0.862 (0.200, 3.721)	0.842
	3	3.359 (1.198, 9.417)	0.021	2.953 (1.051, 8.293)	0.040
pTNM stage	I	1 (reference)			
	II	2.023 (0.240, 17.041)	0.517		
	III	4.081 (0.541, 30.764)	0.172		
NE component 0<NE≤70%		1 (reference)			
NE component 70%<NE<100%/		1.718 (0.756, 3.903)	0.196		
Ki-67 (%)	≤56.21	1 (reference)			
	>56.21	2.696 (0.834, 8.710)	0.097		
Mitotic index	≤37.71	1 (reference)			
	>37.71	1.887 (0.696, 5.118)	0.212		
Adjuvant therapy	N	1 (reference)			
	Y	1.245 (0.449, 3.452)	0.674		

## Data Availability

The data used to support the findings of this study are available from the corresponding author upon request.
